# Trial of a Homœopathist for Starving a Patient to Death

**Published:** 1850-01

**Authors:** 


*Trial of a Homœopathist for starving a patient to death.*—The following singular account is from a London paper of the 10th ult. Mr. H. M. Wakley is, we believe, a son of the well known M. P., Coroner for one division of Middlesex, and proprietor of the *Lancet.*

Last evening, at five o'clock, Mr. H. M. Wakley, deputy-coroner for Middlesex, and a jury of inhabitants of St. Pancras, assembled for the third time at the Perseverence Tavern William street, Hampstead Road, to conclude their inquiry concerning the death of Mr. Richard David Pearce, aged thirty-four, an upholsterer, late of No. 86, Mary street, alleged to have been starved to death, whilst under the homœopathic treatment for cholera, adopted by his brother, Mr. Chas. Thomas Pearce, an unqualified homœopathic practitioner, residing at No. 3, Taunton Place, Regent's Park.

The accused was present throughout the proceedings, accompanied by Mr. Dowding, his solicitor, and the inquiry excited throughout the neighborhood a great deal of interest, especially amongst the medical profession. The following is an epitome of the evidence.

Mrs. Jane Pearce, widow of the deceased, deposed that he was taken ill with diarrhœa and spasms of the stomach, on Saturday, the 8th ultimo. On Sunday morning, the 9th, Mr. Harris, surgeon, of Gower street, was sent for, and on arriving, pronounced deceased to be laboring under cholera. Mr. Harris saw him three times that day, and gave him medicine, and Mr. Charles Thomas Pearce, the accused, came the same evening. When he came, he said he should take deceased under his own care, and she believed he dismissed Mr. Harris. He told her he had taken his brother out of Mr. Harris' hands, and he then gave him a powder, giving her strict injunctions not on any account to give deceased any thing to eat or drink excepting ice water and the medicines he sent. The deceased was under his treatment for ten days previous to his death, which took place on the 18th of September. During the whole of that period, Mr. Pearce, the accused, refused to allow him to have anything to eat. Deceased continually craved for food, and when she appealed to the brother about it, he said if he gave him food she would kill him, and therefore she was afraid to do so. Deceased was continually crying for food, and complained that he was being starved to death. She sent for Mr. Davis, another surgeon, on the evening of the 17th, because her husband became much worse. She did give him some beef tea and some arrowroot, and the day before he died, some toast. He was sick more than three times. He did not get any better under his brother's treatment.

Mr. James Davis, surgeon, of Ampthill Square, deposed that he was called to see deceased on the evening of the 16th ult., and found him in a very low and exhausted condition, apparently for want of nourishment. Deceased was sensible, and told him he had been starved to death by the homœopathic system of medical treatment. He ordered him some brandy and water, and sent him medicine, and when he saw him on the following morning, the 18th, he was dying. Could not say that he considered him laboring under cholera when he first saw deceased. On examining the body he found it much emaciated. The liver, kidneys, lungs, and right side of the heart were congested. The stomach was healthy, but all that was found in it was a small quantity of liquid matter of a brown color, which, on being tested, proved to contain a slight portion of arsenic. In witness' opinion the cause of deceased's death was exhaustion from want of food. Was informed that all deceased had had in shape of food was a little beef tea and some arrowroot. Considered such treatment enough to kill and exhaust any man.

The inquiry was ultimately adjourned till last evening, Mr. Harris, who first attended the deceased, being out of town; and on the re-assembling of the jury—

Mr. Harris was sworn—Finding that the brother of the deceased professed the doctrine of homœopathy, and that he wished deceased to be treated under that system, he told him he could not act in concert with him, and at his request he then gave the care of deceased over to him. He could not say that deceased died of Asiatic cholera. He had cholera when he (Mr. Harris) saw him, and he treated him for it. He did not give up the case because it was hopeless. Having heard the whole of the evidence read over, Mr. Harris said he certainly considered the deceased had not sufficient food to sustain life. On the contrary, he (witness) ordered strong beef tea, arrowroot, brandy and water, and solids, if he could take them.

Mr. Dowding, on the part of the accused, proposed to call Dr. Epps, Dr. Kelsall, and other professors of the homœopathic system who were present, and who, he said, would prove that the treatment of the deceased was skillful and proper.

Dr. Kelsall was sworn—He said he was a professor of homœopathy, and had formerly been a surgeon in the navy. He had had many cases of Asiatic cholera under his care, and he considered the treatment pursued by the accused proper. It was his firm conviction that it was highly improper to give food in cases of cholera. No food whatever should be given from the moment of attack until the patient is convalescent. He had patients under his own care without food for a longer period than the deceased, and he considered it certain death to give a patient any food whatever. To give a patient laboring under cholera arrowroot—you might as well give him sawdust. He had had one hundred patients under his care within the last two months, and he had lost only ten.

Mr. Dowding wished to call Dr. Epps to corroborate this statement, but the jury declined hearing him.

The Coroner, in summing up the evidence, said it was the opinion of the most eminent and educated in the medical profession that the system of homœopathy was a species of humbug and quackery, not founded on medical science. It had been proved that the deceased had died from exhaustion from want of food, and the question was by whose influence the food had been withheld. The coroner then cited the judgments of Lord Lyndhurst and several other judges; showing that if a medical man, whether qualified or not, treated a person unskilfully, and death ensued, he was guilty of manslaughter.

After two hours deliberation. the jury returned a verdict of manslaughter against Charles Thomas Pearce, who was at once committed to Newgate on the Coroner's warrant.—*British American Journal.*

*Results of investigations by a committee of the Royal College of Physicians, London, on microscopial bodies in the intestinal discharges of cholera patients.*

1. Bodies presenting the characteristic forms of the so-called cholera fungi are not to be detected in the air, and as far as our experiments have gone, not in the drinking water of infected places.

2. It is established that, under the terms "annular bodies," "cholera cells," or cholera fungi," there have been confounded many objects of various, and totally distinct natures.

3. A large number of these have been traced to substances taken as food or medicine.

4. The origin of others is still doubtful but these are clearly not fungi.

5. All the more remarkable forms are to be detected in the intestinal evacuations of persons laboring under diseases totally different in their nature from cholera.

Lastly, we draw from these premises the general conclusion, that the bodies found and described by Messrs. Brittan and Swayne are not the cause of cholera, and have no exclusive connection with that disease; in other words, that the whole theory of the disease which has recently been propounded, is erroneous, as far as it is based on the existence of the bodies in question. (Signed),

WILLIAM BALY, M. D.
WILLIAM W. GULL, M. D.

*Medical News.*] *Cholera Sub-Committee.*

